# Patient-specific cerebral 3D vessel model reconstruction using deep learning

**DOI:** 10.1007/s11517-024-03136-6

**Published:** 2024-05-28

**Authors:** Satoshi Koizumi, Taichi Kin, Naoyuki Shono, Satoshi Kiyofuji, Motoyuki Umekawa, Katsuya Sato, Nobuhito Saito

**Affiliations:** 1 Department of Neurosurgery, The University of Tokyo Hospital, 7-3-1Bunkyo-Ku, HongoTokyo, 113-8655 Japan; 2 Department of Medical Information Engineering, Graduate School of Medicine, The University of Tokyo, Tokyo, Japan

**Keywords:** Deep learning, Medical image processing, Magnetic resonance angiography, Aneurysm, Segmentation

## Abstract

Three-dimensional vessel model reconstruction from patient-specific magnetic resonance angiography (MRA) images often requires some manual maneuvers. This study aimed to establish the deep learning (DL)-based method for vessel model reconstruction. Time of flight MRA of 40 patients with internal carotid artery aneurysms was prepared, and three-dimensional vessel models were constructed using the threshold and region-growing method. Using those datasets, supervised deep learning using 2D U-net was performed to reconstruct 3D vessel models. The accuracy of the DL-based vessel segmentations was assessed using 20 MRA images outside the training dataset. The dice coefficient was used as the indicator of the model accuracy, and the blood flow simulation was performed using the DL-based vessel model. The created DL model could successfully reconstruct a three-dimensional model in all 60 cases. The dice coefficient in the test dataset was 0.859. Of note, the DL-generated model proved its efficacy even for large aneurysms (> 10 mm in their diameter). The reconstructed model was feasible in performing blood flow simulation to assist clinical decision-making. Our DL-based method could successfully reconstruct a three-dimensional vessel model with moderate accuracy. Future studies are warranted to exhibit that DL-based technology can promote medical image processing.

**Graphical Abstract:**

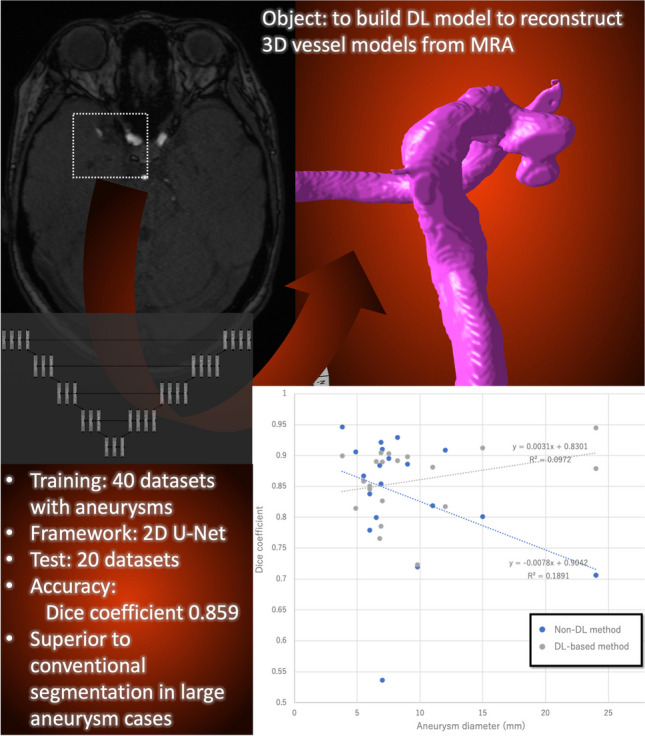

## Introduction

With the advancement of computer technology, patient-specific medical images have been used to construct 3D computer graphics through registration between different imaging modalities [1, 2]. In neurosurgery and neuroradiology, these models not only provide better visualization and understanding of brain tumors and cerebrovascular disease, but also enable computational fluid dynamics (CFD) simulation of cerebral blood flow and simulation of treatment using ex vivo models made from 3D printers [3–7]. These 3D models are expected to help experts and trainees share disease comprehension and learn complex neurosurgical procedures.

However, compared to the improvement of computer power, 3D model reconstruction still requires manual work. While the burden has been reduced with the advancement of 3D fusion image technologies with commercially available software such as GRID (Kompath, Tokyo, Japan) and Brainlab Elements (Brainlab AG, Munchen, Germany), individual models often need manual correction before practical application. These procedures often require special techniques from experienced engineers, and the application of patient-specific 3D vessel models is still limited to research purposes.

Meanwhile, artificial intelligence technology has developed in the field of diagnostic radiology. Specific organ segmentation from non-labeled medical images is already possible with high accuracy, and it helps radiologists with diagnosis and decision-making [8, 9]. Recent literature suggests that the reconstruction of 3D organ models, including the cardiovascular system and lungs, from patient-specific medical images has become possible with deep learning (DL) technology [10, 11]. However, to the best of our knowledge, no studies have yet explored the application of DL for the 3D reconstruction of intracranial vessels. In this study, we applied DL to construct 3D cerebral artery models from patient-specific medical images and validated if these techniques can promote the wider use of 3D imaging for medico-engineering purposes.

## Materials and methods

### Study design and participants

This study was approved by the institutional review board of our hospital (certificate number 2020179NI). We retrospectively reviewed time-of-flight magnetic resonance angiography (TOF-MRA) performed at our department between January 2018 and December 2021 in 40 patients with unruptured intracranial aneurysms in their internal carotid arteries. These were used as training datasets. We used a 3-T scanner (Magnetom Skyra; Siemens Healthcare K.K., Tokyo, Japan) with a 32-channel head/neck array coil for the TOF-MRA. Image acquisition parameters were as follows: repetition time, 42 ms; echo time, 3.6 ms; flip angle, 18°; field of view, 22 cm; voxel size, 0.5 × 0.5 × 0.5 mm; matrix size, 412 × 448.

**Fig. 1:**
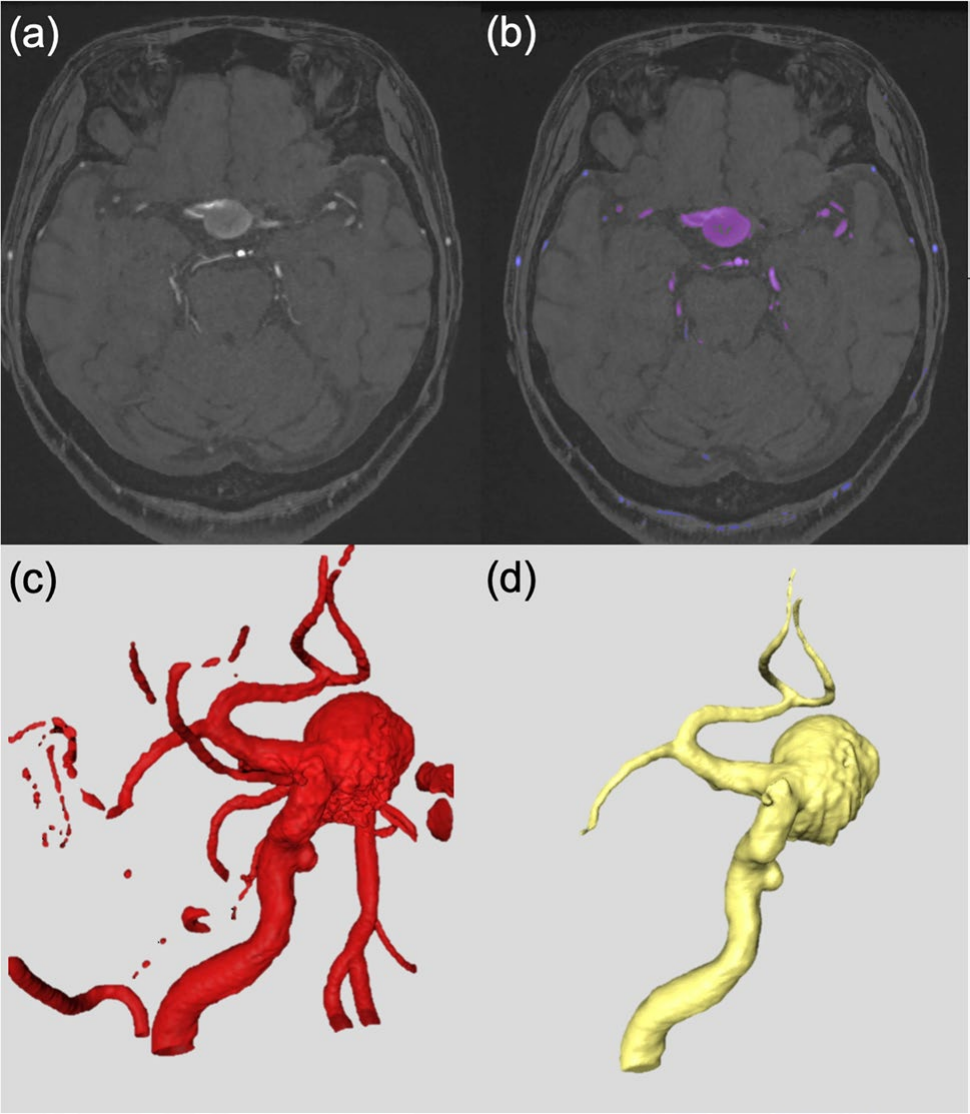
The previously suggested non-DL method of vessel segmentation and 3D vessel model reconstruction. **a** TOF-MRA DICOM images are incorporated into image segmentation software. **b** The artery of interest is segmented using a single threshold and region-growing method. **c** The 3D vessel models are constructed using the segmented vessels. **d** Manual correction is added to generate final vessel model. Procedures such as segmentation of lower velocity areas, undesired vessels removal, and disconnection of adjacent arteries running nearby are necessary processes before actual application

To perform supervised learning, processing of the TOF-MRA in the Digital Imaging and Communication in Medicine (DICOM) format was performed using Amira version 2019.4 software (Thermo Fisher Scientific, Hillsboro, OR). Regions starting from the petrous segment of the internal carotid artery to the proximal part of the second portion of the middle (and anterior, if any) cerebral artery were trimmed. To account for the spatial resolution limit of TOF-MRA, the model was resampled to a 0.15 mm voxel size with a “resample” function of Amira software. The median slice number per case in the training dataset was 410. For the labeling of the intracranial artery, 50% of the maximal signal intensity of the intracranial artery was set as the segmentation threshold to extract vessel morphology [12]. However, volume data selected using a single threshold and the region-growing method cannot function as a practical vessel model. As such, some manual corrections were necessary. These corrections were as follows: Extraction of the small branching artery below 1 mm in diameter, isolation of the fused arteries running closely but unnatural considering the normal anatomy, segmentation of the slow velocity region not labeled by region growing method. Those ground truth vessel models were created by a single author of this study (SK). The model’s accuracy was confirmed by referencing other imaging studies, such as 3D rotational angiography and computed tomography angiography. It was also verified by another author (TK) who is experienced in computer-based medical image processing. The visual summary of the previously suggested 3D model reconstruction method (non-DL method) is shown in Fig. 1.

### DL segmentation and vessel modeling

**Fig. 2:**
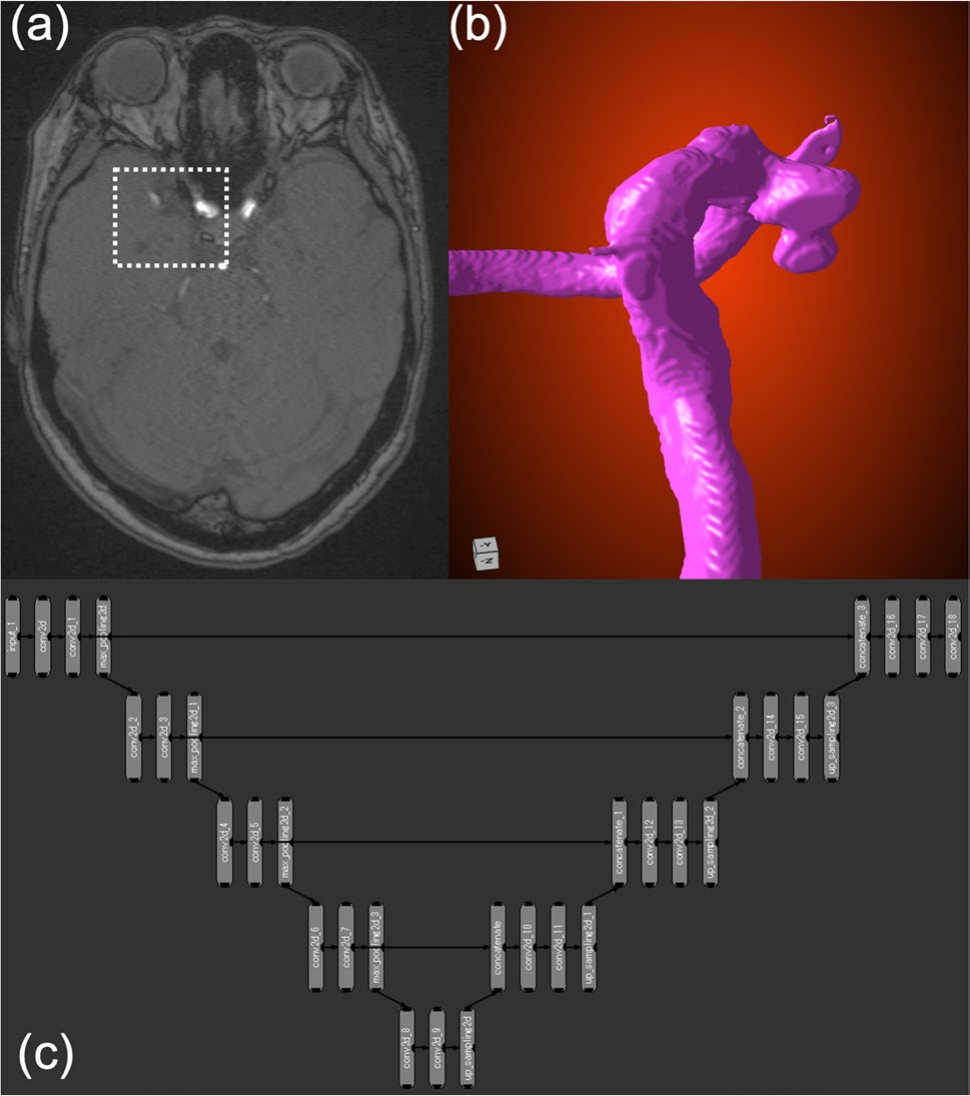
The proposed method of generating a three-dimensional vessel model from TOF-MRA. **a** TOF-MRA DICOM images are incorporated into DL image segmentation software. The dotted lines show the region of interest cropped. **b** The prepared images are put into 2D-U-net-based DL model to construct vessel models including the internal carotid artery and the aneurysm. **c** Final 3D models are generated

A dataset consisting of TOF-MRA DICOM images and a 3D vessel model constructed using a non-DL method was prepared for use in supervised DL. The DL was performed using the DL tool implemented in Dragonfly version 2021.3 (maxnet, Tokyo, Japan), a graphical user interface-based software made for medical image segmentation and modeling. The 2D U-net, a DL framework commonly used in medical image segmentation, was employed. The DL parameters used were as follows: patch size 128, stride ratio 0.8, batch size 64, and epoch number 200. Augmentation of the original training data was not performed in this study due to constraints on computer power. The Adadelta algorithm was used to optimize the learning rate [13]. Eighty percent of the dataset was used for training, while the remaining 20% was used for internal validation. These internal validation cases were used as a reference to enhance the accuracy of the deep learning (DL) model during each epoch, utilizing binary cross entropy as a loss function. A visual summary of the DL-based 3D vessel model reconstruction method is shown in Fig. 2.

### Assessment of the constructed vessel model and its application

DL-generated vessel models were compared to the vessel models constructed using the non-DL method. To assess the quality of the DL-generated models, 20 MRAs of patients separate from the training data (test data) were used. The imaging parameters of MRAs and the resampling method used for preparing the test data were identical to those used in preparing the training data. The time taken to generate the 3D vessel model from resampled MRA data was measured for these test data. The Dice coefficient was used to quantify the consistency of the generated model. The 3D vessel model defined with a single threshold and generated with DL was compared to the model segmented with the non-DL method.

### CFD analysis

To assess the feasibility of DL-generated vessel models, CFD simulations of blood flow were performed using a commercially available finite volume solver (SCRYU/Tetra for Windows Version 14; Software Cradle Co, Tokyo, Japan), as previously described [5, 6]. The DL-generated vessel models were converted to surface models (.stl) using the exporting function of Dragonfly. From these models, tetrahedral elements with a maximal size of 0.125 mm were created, and three boundary layers of prismatic elements were inserted in each case to ensure finer resolution and resolve high-velocity gradients close to the endothelial layer. To guarantee fully developed velocity profiles within the region of interest, all inlets were extruded by 10 times the corresponding diameter.

The velocity fields were determined under the governing equations of continuity and Navier–Stokes. Spatial distributions of pressure were determined by solving the Poisson equation of pressure to complete the velocity fields. Boundary conditions were defined using specific parameters. Blood was assumed to be an incompressible Newtonian fluid with a specific gravity of 1053 kg/m^3^ and a viscosity of 4.0 × 10^−3^ N·s/m^2^. The viscoelastic properties of the vessel wall were neglected, and a rigid wall with a no-slip condition was assumed. The inflow rate of the internal carotid artery at its petrous portion was defined based on its diameter [14]. A static pressure of 0 Pa was assumed for all outlet vessels. The computed velocity and geometry information were analyzed using the postprocessor within the simulation software. The inflow volume to each aneurysm was calculated from the blood flow simulation, as low inflow volume calculated on preoperative computer simulation is highly predictive of aneurysm occlusion after flow diverter stenting, as suggested by Zhang et al. in their meta-analysis [15].

### Statistical analysis

All statistical analyses were performed using R version 4.1.1 (The R Foundation for Statistical Computing, Vienna, Austria; [http://www.R-project.org/] (http://www.r-project.org/)). The normality of the continuous variable distribution was assessed using the Shapiro–Wilk test. Data that are normally distributed are expressed as mean ± standard deviations. If the data are not normally distributed, they are expressed as median [interquartile range]. Categorical data are expressed as the number of subjects (percentage of the total). To compare these variables between the groups, Fisher’s exact test, Mann–Whitney *U* test, and *T* test were used, respectively. All statistical tests were two-sided, and a *P*-value < 0.05 was considered statistically significant.

## Results

Table 1 summarizes the characteristics of aneurysms in both the training and test data. Age, sex, and aneurysm size did not differ significantly between the two sets. Most aneurysms in both sets were between 5 and 15 mm in size. However, there were more cavernous aneurysms in the training data (14 [35%] vs. 1 [5%], *P* = 0.012).

**Table 1:** Basic characteristics of the aneurysm data included in the study. Continuous variables are expressed as mean ± standard deviation if the data is normally distributed, or as median [IQR] if the data are not normally distributed. Categorical data are expressed as the number of subjects [percentage of the total].* < 0.05

	Training data	Test data	*P*
*N*	40	20	-
Male	3 [7.5%]	4 [20%]	1
Age (y.o.)	63 ± 14	62 ± 13	0.70
Aneurysm size (mm)	9.5 [6.5–15]	7.0 [6.4–10.0]	0.37
Cavernous location	14 [35%]	1 [5%]	0.012*
Slice number	410 [347–478]	409 ± 36	0.95

**Fig. 3:**
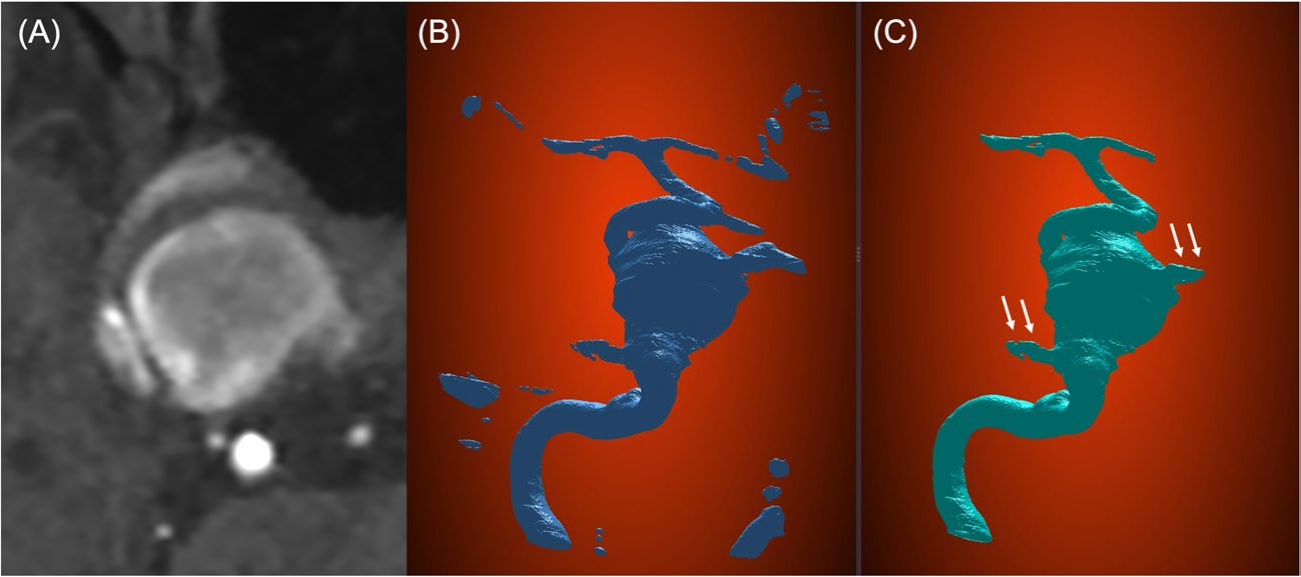
An example of 3D vessel model reconstruction using the proposed method. **A** The original TOF-MRA shows the right internal carotid cavernous aneurysm, which is 21 mm in size. Note the heterogenicity of the signal intensity inside the aneurysm. **B** The original vessel model generated from the DL model. **C** The regions remote from the areas of interest are removed from the previous model. The aneurysm and the artery are adequately segmented with minimal noises close to the parent artery and the aneurysm (white arrows)

**Fig. 4:**
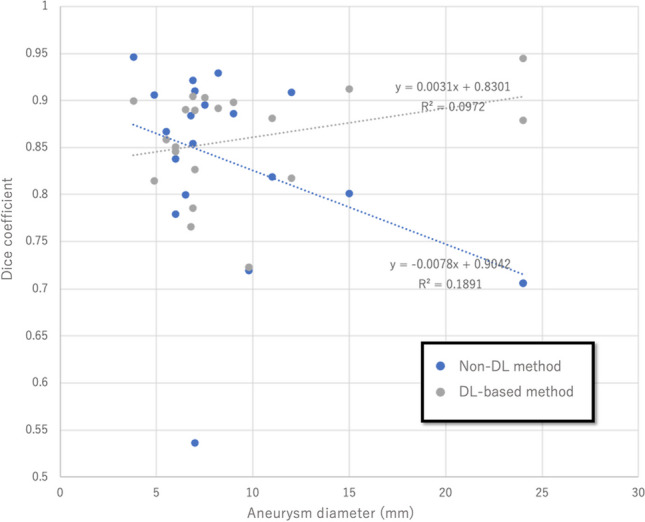
The relationship between vessel model accuracy and aneurysm size. In single threshold segmentation, the accuracy of the 3D vessel model decreases as the aneurysm size increases. However, this tendency is not observed in the DL-based segmentation

The segmentation of the internal carotid artery and aneurysm was successful in all the training and test datasets. After DL, the loss function (binary cross entropy) in the validation dataset was 0.008. In the test dataset, the average time taken to segment the vessel using DL was 56 ± 5.2 s, and the average Dice coefficient of the DL-generated model was 0.86 ± 0.055. An example of DL-based vessel segmentation is shown in Fig. 3**.** In contrast, the average Dice coefficient of the vessel models defined with a single threshold was 0.86 [0.79–0.91]. The consistency of the DL-generated 3D vessel model was not statistically different from that of the single threshold model (*P* = 0.62, Mann–Whitney *U* test). Notably, the Dice coefficient of the single threshold model tended to decline as the aneurysm grew, but this tendency was not present in the DL-generated models (Fig. 4).

**Fig. 5:**
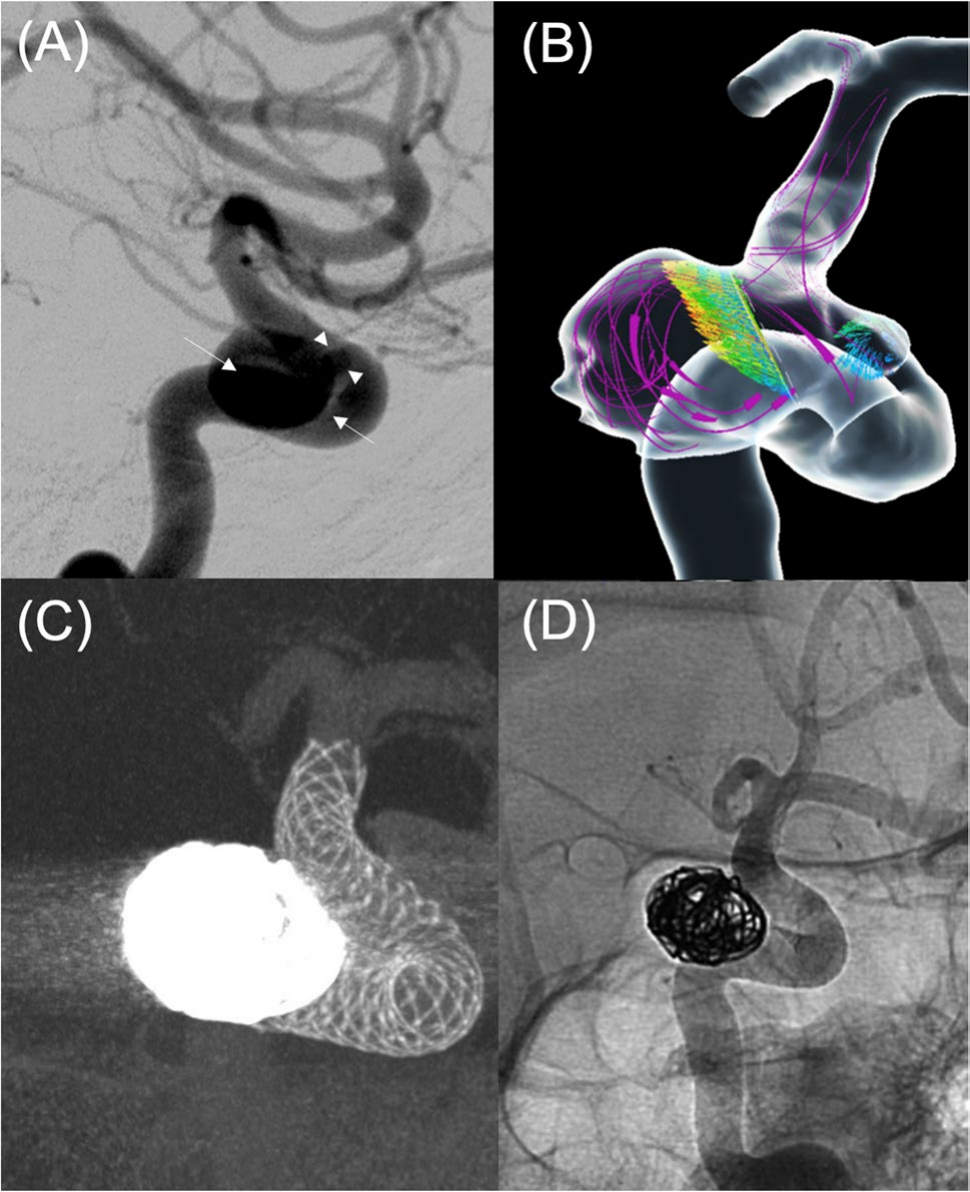
Preoperative digital subtraction angiography (**A**), CFD analysis (**B**), and endovascular treatment (**C**, **D**) for unruptured intracranial aneurysms located at the C2 and C3 portions of the left internal carotid artery. **A** The larger C2 aneurysm (white arrows) was 9 mm in diameter. The smaller aneurysm (white arrowhead) was 2 mm in diameter. **B** A schematic of the CFD analysis. The pink arrows show the streamlines of the blood flow from the proximal internal carotid artery. The inflow to each aneurysm was shown with colored vector arrows, with the colormap from blue to red representing the absolute value of each vector. Compared to the smaller C3 aneurysm, more blood inflow into the larger C2 aneurysm can be recognized. **C**, **D** Assisted by the CFD findings, the aneurysms were treated using a flow diverter stent covering the two aneurysms. To promote occlusion of the larger C2 aneurysm, coils were additionally inserted into the C2 aneurysm. Note that the blood flow into the C3 aneurysm almost disappeared immediately after the flow diverter placement

In all 60 cases, it was technically feasible to perform CFD analysis using a DL-generated vessel model. For a representative patient with multiple unruptured intracranial aneurysms (9 mm at the left internal carotid artery C2 portion and 2 mm at the C3 portion), a preoperative CFD assessment was conducted (Fig. 5). The simulation revealed that the larger C2 aneurysm had a relatively high blood inflow (2.0 mL/s), whereas the inflow volume of the smaller C3 aneurysm was scarce (6.4 × 10^−5^ mL/s) in relation to the blood flow volume of the parent internal carotid artery (3.5 mL/s). In the treatment, a flow diverter stent was placed to cover the two aneurysms, and with the help of the CFD simulation findings, detachable coils were additionally inserted inside the larger C2 aneurysm to promote its occlusion.

## Discussion

In this study, we developed a DL model to extract 3D vessel morphology from TOF-MRA. Although the number of subjects in the training dataset was not large (40 cases), our model was able to segment intracranial vessels with moderate accuracy, and 3D vessel models could be constructed with simple removal of remote regions. The generated model exhibited non-inferior accuracy to the single threshold segmentation model. Especially, the DL-based model exhibited higher performance as the aneurysm size in the test data increased.

Conventionally, DL technology has already been reported to be useful in the diagnosis and decision-making of cerebrovascular diseases. For example, Joo et al. reported a DL algorithm that can automate intracranial aneurysm detection on MR angiography with high diagnostic performance [16]. Olive-Gadea et al. developed a DL-based software to identify large vessel occlusion on non-contrast computed tomography [17]. Meng et al. proposed an end-to-end artificial intelligence platform for the management of large vessel occlusions [18]. The current study has its novelty in exhibiting that DL can be useful for medical image segmentation and its three-dimensional processing.

To extract vessel morphology from TOF-MRA, various methods have been proposed. The most representative one is to use half-width, in which 50% of the maximal signal intensity is defined as the threshold to segment intracranial arteries [19]. However, in the aneurysm sac, the direction of blood flow is not uniform, the blood flow velocity is relatively low, and the flow can sometimes be turbulent. For those reasons, it can often be difficult to define aneurysm wall morphology with a simple threshold and region-growing method. To resolve those difficulties, solutions using multiple thresholding were proposed and exhibited satisfactory results [20, 21]. In conventional non-DL-based vessel reconstruction, the anatomical insights of neuroradiologists or neurosurgeons are needed to create a clinically useful vessel model. However, computer-based vessel segmentation and 3D reconstruction are not familiar techniques for most clinicians. Depending on the case, the non-DL-based vessel model reconstruction used in this study typically takes between 1 and 3 h. In contrast, once the learning model is established, the DL-based 3D vessel model reconstruction proposed in this study usually only takes several minutes. It is also easily accessible for clinicians unfamiliar with DL technical details. Therefore, DL-based vessel model reconstruction has the potential to advance the medical-engineering application of cerebral vessel models including CFD analysis and 3D printing.

There are several preceding studies applying DL to intracranial vessel segmentations. Garcia et al. constructed a model for brain vessel segmentation model in 3D rotational angiography with arteriovenous malformations [22]. They used 3D U-net as a DL framework and concluded that their model achieved a better characterization of the malformation topology and morphology. Livne et al. created a 2D-U-net-based model to segment intracranial vessels with stenoocclusive disease from TOF-MRA [23]. They exhibited that the combination of 2D U-net and TOF-MRA was sufficient to construct the vessel model. The DL framework and the imaging modality of the training data should be selected in relation to the required topographical accuracy of the 3D vessel model.

To the best of our knowledge, the current study is the first one to focus on the segmentation of intracranial arteries with cerebral aneurysm. Although the reported prevalence of unruptured intracranial aneurysm is as high as 2–3% in the general population [24], our current understanding of the natural history of unruptured intracranial aneurysm is still insufficient in predicting its growth and rupture for each specific aneurysm [25–27]. If novel medico-engineering innovation like blood flow analysis and treatment simulation can assist clinical decision-making, it will be of great help to both clinicians and patients. Future studies are warranted to validate if DL-based vessel model reconstruction has the potential to help such medico-engineering innovations in the management of intracranial aneurysms.

There are several limitations to this study. Firstly, the number of subjects in the training dataset was not large, which may have limited the accuracy and external validity of the DL model. To promote the wider use of the DL model in various clinical settings, robustness assessments and confirmations through cross-validation or external validation are essential. This study serves as a preliminary step, confirming the applicability of DL-based 3D vessel reconstruction in a relatively simple condition. Future studies should aim to verify its effectiveness across diverse input data. Secondly, the use of the Dice index in assessing the vessel model might not be wholly adequate. It provides a practical index for quantitative evaluation of the model’s overall accuracy, but it is less effective in evaluating accuracy in and around the aneurysm. To overcome this, we conducted additional CFD simulations with the DL-segmented vessel model and included a representative case in the manuscript. However, this approach still has limitations in fully assessing the constructed vessel model. Comparing the CFD results of the ground truth vessel model and the DL-generated vessel model could be a future method to assess the practical applicability of the DL-generated vessel model. The feasibility of the DL-generated vessel model for medical engineering applications needs to be validated in future studies with a larger number of cases. Finally, we did not evaluate deep learning (DL) parameter optimization. The DL parameters used in this study were chosen based on our computational capabilities. Without computational constraints, a larger batch size could expedite DL. To build a DL model that captures subtle anatomical features near the aneurysm, a smaller patch size and stride ratio would be ideal. However, this requires more computational power. Regarding the number of epochs, a sufficiently large number is crucial for achieving learning convergence and stabilizing the model’s performance. Fine-tuning these parameters should be addressed in the future.

## Conclusion

This study demonstrates that DL-based segmentation can serve as an alternative solution for extracting desired components from patient-specific medical images. Future studies are needed to show that DL-based technology can reduce the burden of medical image processing and promote wider application of patient-specific 3D models in medico-engineering.
